# Histomorphometric and vascular changes in equine endometrium after the infusion of conceptus fragments

**DOI:** 10.1590/1984-3143-AR2020-0006

**Published:** 2020-06-29

**Authors:** Cesar Augusto Camacho, Maria José Estradé, Nicolás Cazales, Jorge Emilio Caballeros, Sandra Mara Fiala-Rechsteiner, Adriana Pires Neves, Rodrigo Costa Mattos

**Affiliations:** 1 Laboratório de Reprodução Animal, Faculdade de Veterinária, Universidade Federal do Rio Grande do Sul, Porto Alegre, RS, Brasil; 2 Facultad de Veterinaria, Universidad de la República, Montevideo, Uruguay; 3 Historep, Instituto de Biologia, Universidade Federal de Pelotas, Pelotas, RS, Brasil; 4 Universidade Federal do Pampa, Dom Pedrito, RS, Brasil

**Keywords:** ultrastructure, histological, vascularization, uterus

## Abstract

This experiment aimed to verify if the proteins present in a 13^th^ day conceptus induce changes in the equine endometrial ultra-structure, histology, and vascularization, two days after its infusion. Ten healthy cyclic mares were used. Once estrus was confirmed, mares were examined daily to detect ovulation (day 0). After ovulation, mares were examined daily until day seven by transrectal palpation and B-mode and Doppler ultrasonography. In this first cycle, intrauterine biopsies were collected at day seven after ovulation, constituting the Cyclic group (n = 10). In the second cycle, the same mares daily were examined until ovulation was detected. After ovulation, mares were examined daily by transrectal palpation and B-mode and Doppler ultrasonography until day 7. On day 5, after ovulation, fragments from previously collected 13-day-old concepti were infused into the uterus of each mare. Intrauterine biopsies were collected at day 7 in all mares (n = 10), constituting the Fragment group. The percentage of ciliated and flattened cells decreased in the Fragment group. Protruded cells, superficial and intraglandular secretion, glandular lumen and diameter, blood vessel diameter, endometrial vascularization, and immune cells were higher in the Fragment group than in the Cyclic group. In summary, proteins of 13^th^ day equine conceptus fragments infused at day five after ovulation signaled histological and vascular changes in the endometrium at the 7^th^ day after ovulation.

## Introduction

The uterus is a dynamic physiological system in which cellular proliferation and differentiation occur during pregnancy (Das, 2009). A swift endometrial adaptation occurs when the embryo enters the uterus. Ultra-structural and histological changes of the endometrium were reported in pregnant mares from the 7^th^ day after ovulation, compared to cyclic mares (Camozzato et al., 2019). In pregnant mares, the number of leukocytes increased in association with a local immunological response (Keenan et al., 1987). The endometrium of inseminated mares’ present ultra-structural and histological changes with an increase of lymphocytes on the 5^th^ day after ovulation before the embryo enters the uterus (Caballeros et al., 2019). Embryo presence was associated with increased blood flow and vascular perfusion from the uterine artery on the 11^th^ day after ovulation (Bollwein et al., 2003) and to local vascular changes related to embryo localization (Silva et al., 2005).‬‬‬‬‬‬‬‬‬

The horse is one of the few domestic species in which the conceptus-derived pregnancy recognition signal has not been identified (Klein and Troedsson, 2011a). Persistence of the corpus luteum is seen in a certain percentage of non-pregnant mares after the introduction of a glass marble (Nie et al., 2003) or fluid-filled rubber ball (Rivera del Alamo et al., 2008) into the uterine lumen in the first days after ovulation. Concepti obtained before day 14 after ovulation release a characteristic pattern of proteins, and one or more of these proteins may be involved in the anti-luteolytic mechanism (McDowell et al., 1990), which prolongs the lifespan of the corpus luteum. These results suggest that the embryonic signal for maternal recognition of pregnancy in the horse might be at least in part secretory rather than mechanical (Aurich and Budik, 2015).

Conceptus fragments have been used as study models to improve the understanding of anti-luteolytic control, maternal pregnancy recognition, pregnancy maintenance, and the maintenance of corpus luteum lifespan in mares (Ball et al., 1989) and ruminants (Rowson and Moor, 1967; Camous et al., 1984; Heyman et al., 1987). Culture systems with trophoblastic vesicles and monolayers of equine uterine epithelial cells have been used to examine events associated with maternal recognition of pregnancy in the mare, determining cell-cell communication (Brady et al., 1993).

It is hypothesized that proteins present in conceptus fragments infused into the equine uterus five days after ovulation will induce changes in the equine endometrial ultra-structure, histology, and vascularization that can be detected at day seven after ovulation. This experiment aimed to verify if the proteins present in a 13th-day conceptus induce the changes mentioned above in the equine endometrium, two days after its infusion.

## Methods

### Animals

The study was performed in the southern hemisphere-breeding season. Ten healthy cyclic Quarter Horse-type mares (mean age 6.8, ranging between 4 to 10 years old), weighing 450-550 kg were used. Mares were kept in natural pastures with free access to mineral supplementation and water. Throughout the experiment, mares remained healthy, with an average body condition score of 3.5 (scale 1 to 5) (Malschitzky et al., 2001).

Mares were examined for reproductive soundness by evaluation of perineal conformation, transrectal palpation, ultrasound of the genital tract (Sonoscape S8V, China), and endometrial biopsy. Only cyclic and clinically normal mares, with endometrium classified as category I or IIA (Kenney and Doig, 1986) and without evidence of endometritis, were selected. All the mares had previously foaled.

### Experimental design

Mares’ reproductive tracts were routinely examined by transrectal palpation and B-mode ultrasonography until estrus was detected. Once estrus was confirmed (ovarian follicle > 35 mm in diameter and marked uterine edema), mares were examined daily to detect ovulation, considered day 0. After ovulation, mares were examined daily by transrectal palpation and B-mode and Doppler ultrasonography until day 7. In this first cycle, endometrial biopsies were collected at day seven after ovulation in all mares (n = 10). These mares constituted the Cyclic group (CG).

In the second cycle, the same mares were daily examined until ovulation was detected (day 0). After ovulation, the mares were examined daily by transrectal palpation and B-mode and Doppler ultrasonography until day 7. On day 5, after ovulation, fragments from concepti recovered previously from pregnant mares were infused into the uterus to each mare. Intrauterine biopsies were collected at day 7 in all mares (n = 10). These mares constituted the Fragment group (FG).

### Conceptus fragments

Conceptus fragments were obtained from previous embryo collections performed on day 13 after ovulation of other mares and snap-frozen in liquid N2 with 2.5 mL of Ringer solution. Before their infusion in the FG mares, concepti were thawed at room temperature, where they collapsed due to their osmolality and size. Protein denaturation can be prevented by rapid cooling to temperatures as low as -70 °C, maintaining its structure sufficiently well and enabling normal enzymatic catalysis to proceed (Fahy and Wowk, 2015). Thawed concepti were divided equally into two parts. Each part was transferred to a Petri dish, diluted with Ringer solution to 2 mL, homogenized, and infused to each mare (n = 10). Infusion of the fragments was carefully performed at day five after ovulation with a nonsurgical trans-cervical procedure, using a standard artificial insemination pipette protected with a sterile outer chemise. Conceptus fragments (2 mL) were deposited into the uterine body.

### Endometrial biopsies

Two biopsies were obtained per mare on day seven after ovulation, one from the dorsal wall of each uterine horn, close to the bifurcation, at each cycle. Each biopsy sample from the Cyclic (n = 20) and Fragment (n = 20) groups was bisected and stored separately in 5-mL tubes containing 2.5% glutaraldehyde solution (0.01 mol/L phosphate buffer, pH 7.3) for scanning electron microscopy (SEM) studies and 4% buffered paraformaldehyde solution for light microscopy and morphometry studies.

### SEM

SEM gives a three-dimensional representation of tissue surface ultrastructure, exhibiting the epithelium of the uterus, which is folded into long ridges and troughs. The samples for SEM were dehydrated through a graded series of acetone. The tissue was dried in a critical point drier (CPD030 – Balzers) using carbon dioxide. The dried pieces were attached to stubs with double-sided adhesive tape and were sputter-coated with gold/palladium (Sputter Coater SCD050 – Balzers). Samples were scanned and photographed with a JEOL (JSM 6060) digital scanning electron microscope. For image analysis, five pre-defined squares were superimposed to SEM images. For each mare in each group, five images of each horn were evaluated, totaling 100 areas/group. Ciliated cells, polygonal microvilli secretory cells (protruded and flattened), and apical blebs were counted, and the percentage of area occupied by the different cells and by the superficial secretion was calculated. Quantification was performed using ImageJ analyzing software (ImageJ, National Institutes of Health, Maryland, USA).

### Light microscopy

Light microscopy provides transverse images of the tissue, showing structures in the stratum compactum and spongiosum of the endometrium, including the luminal epithelium, endometrial glands, stroma, and blood vessels. Samples conserved in formalin were processed to be included in paraffin. Paraffin blocks were cut with an automated microtome (Leica, RM165) at 5-µm thickness, adhered in histology slides, and kept on a 60 °C incubator. After deparaffinization, cuts were stained using routine techniques for tissue samples (Tolosa et al., 2003), using hematoxylin and eosin (H-E) staining. All sample slides were analyzed under light microscopy by an experienced pathologist blinded from any information.

The morphological features were photo-documented using an optical microscope (DM500, Leica Microsystems GmbH, Germany) with an attached capture camera (ICC500 HD, Leica Microsystems GmbH, Germany), and an image acquisition software (LAS EZ, Leica Microsystems GmbH, Germany). Measurements and counting were done using the ImageJ image analyzing software (ImageJ, National Institutes of Health, Maryland, USA).

The following measures were performed in both uterine horns:

- Intraglandular secretion: quantified using the mean of the two largest diameters of the secretion inside ten randomly selected spherical glands at 400x magnification from each horn (n = 20/mare);
- Glandular diameter: obtained using the mean of 2 perpendicular diameters of each gland (from a basement membrane to the opposite one). Measured in 10 randomly selected spherical stratum spongiosum glands at 400x magnification from each horn (n = 20/mare);
- Glandular lumen: measured in the same way as the previous variable, measuring the space between the apical membranes of the epithelial cells, recorded in 10 randomly selected spherical stratum spongiosum glands at 400x magnification from each horn (n = 20/mare);
- Glandular density: 3 fields from each horn (n = 6/mare) were observed at 100x magnification to evaluate glandular density in both the stratum spongiosum and the stratum compactum, counting the glands per stratum in each field;
- Height of the glandular epithelium: measured from the basal lamina of the cells to the apical membrane of the cells recorded at 400x magnification in 10 randomly selected spherical stratum spongiosum glands from each horn (n = 20/mare);
- Height of the luminal epithelium: measured from the basal lamina to the apical membrane of the cells, recorded at 400x magnification in 5 randomly selected fields from each horn (n = 10/mare);
- Immune cells: lymphocytes, eosinophils, and neutrophils counted in 5 fields from each horn (n = 10/mare) at 1,000x magnification in the stratum spongiosum and the stratum compactum;
- Blood vessel diameter: measured in 5 fields from each horn (n = 10/mare) at 1,000x magnification. Total blood vessel area was calculated by multiplying the vessel diameter by the number of vessels observed in the studied field.

An average of the records of each variable for each uterine horn was calculated.

### Doppler uterine hemodynamics

The analysis of hemodynamics was performed using a Doppler ultrasound (SonoScape^®^ model S8V) with a transrectal linear probe (5-10 MHz) in Power Doppler and Spectral Doppler modes, using a frequency of 7.2 MHz, a filter of 100 HZ and 5.5 cm/s of flow detection.

#### Power Doppler

**Figure 1:**
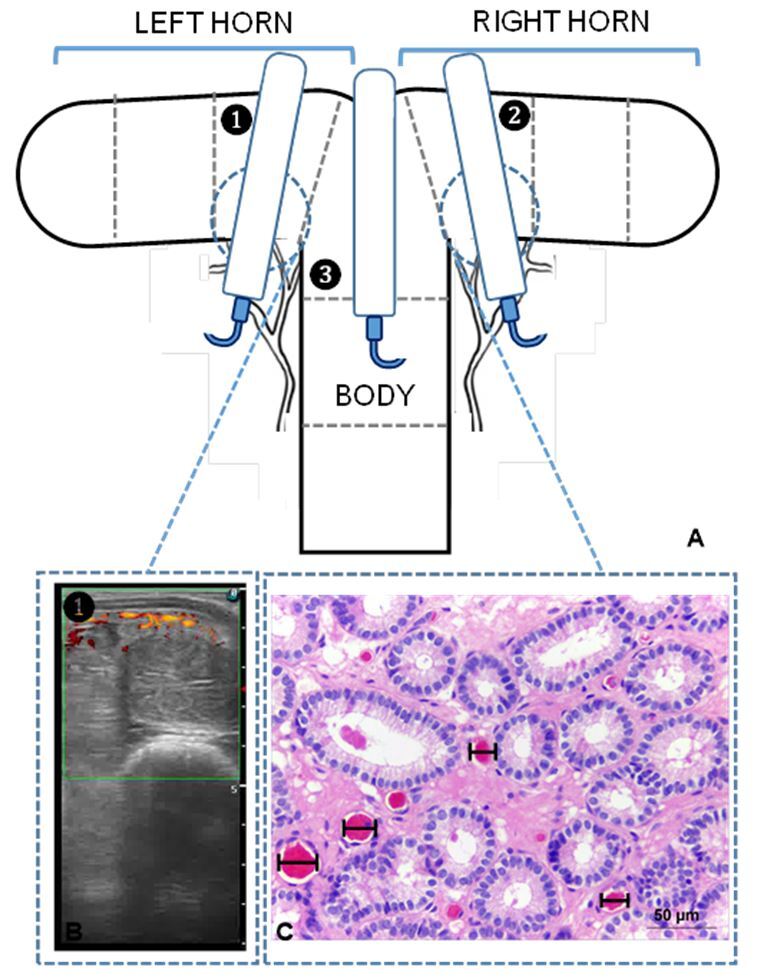
(A) Diagram of the transrectal placement of a linear-array ultrasound transducer, showing the spatial relationships between the uterine horns and transducer. The numbers show the 3 uterine segments used in the experiment; (B) Ultrasonography image. The colored spots are Power-Doppler indicators of blood flow; (C) Histological section of an endometrial biopsy; black bars show blood vessel diameter.

Vascular perfusion of the endometrium was evaluated using the Power Doppler mode. The transducer was placed over the uterine body and over a cross-section of the middle segment of each uterine horn (Figure 1). Vascular perfusion was estimated subjectively and objectively.

The subjective evaluation scored the extent of colored areas in the endometrium for 30 seconds, and videos were recorded as Windows Media Video (WMV). The score ranged from 1 to 4, with 1 being the absence of perfusion and 4 being maximal perfusion (Silva et al., 2005)‬‬‬‬‬‬‬‬‬‬‬‬‬‬‬‬‬‬‬‬‬‬‬‬‬‬‬‬‬‬‬‬‬‬‬‬‬‬‬‬‬‬.

The objective evaluation was performed by offline measurement of the area and intensity (pixels) of the blood flow of the endometrium to provide a quantitative measure of the extent of perfusion and relative velocity (Ginther, 2007). The videos mentioned above were transformed and saved as GIF images using Total Video Converter 3.11 (Copyright^©^ EffectMatrix Ltd., Company from 2002 - 2016). From each group, five still images from cross-sections of the middle segment of horns and uterine body from each mare were used for determination of area and intensity, and the average was used in the analyses. The measurement of area and intensity were performed with ImageJ 1.31v software (National Institutes of Health, Maryland, USA) (Silva et al., 2005)‬‬‬‬‬‬‬‬‬‬‬‬‬‬‬‬‬‬‬‬‬‬‬‬‬‬‬‬‬‬‬‬‬‬‬‬‬‬‬‬‬‬.‬‬‬‬‬‬‬‬‬‬‬‬‬‬‬‬‬‬‬‬‬‬‬‬‬‬‬‬‬‬‬‬‬‬‬‬

#### Spectral Doppler

Both the left and right uterine arteries were examined transrectally as previously described (Bollwein et al., 1998) in all the mares in each group. The transrectal pulsed Doppler ultrasound examinations were always performed once daily between 07:00 and 10:00 h and lasted approximately 20 min for each mare.

All blood flow velocity waveforms were displayed online and saved. Following the collection of all data, the Doppler calculations were performed off-line using three similar consecutive flow velocity waveforms with maximum end-diastolic frequency shift. Analysis was based on the envelope of the Doppler shift spectrum.

The following parameters were measured in the Spectral Doppler:

- Time-averaged maximum velocity (TAMV): an average of the maximum velocity values (upper surface of spectrum) over the time of a cardiac cycle;
- Pulsatility index (PI): expression of the extent of the difference between the peak systolic velocity and end-diastolic velocity in the TAMV, indicating a negative relationship between the extent of pulsatility in the tissues and the extent of the vascular perfusion. Increasing PI values indicate decreasing perfusion of the distal tissues;
- Resistance index (RI): expression of the extent of the difference between the peak systolic velocity and end-diastolic velocity in the peak systolic velocity, indicating a negative relationship between the extent of resistance in the tissues and the extent of the vascular perfusion. Decreasing RI values indicate increasing blood perfusion.

Changes of the RI and PI are highly correlated. Data was collected with the Doppler ultrasound software (SonoScape^®^ model S8V).

### Statistical analysis

Variables were evaluated using the Statistical Analysis System (SAS, Cary, NC, USA). Percentage of the area occupied by ciliated cells, polygonal microvilli secretory cells (protruded and flattened), apical blebs, superficial secretion, glandular diameter, glandular lumen, glandular density, height of glandular and luminal epithelium, the diameter of endometrial blood vessels, total blood vessel area, immune cells, subjective vascular perfusion, objective area, and intensity of blood-flow, TAMV, RI, and PI, were considered as dependent variables. Groups (Cyclic or Fragment) and uterine horn (ipsilateral or contralateral to corpus luteum) were considered as independent variables.

Variables were evaluated for normality using the PROC UNIVARIATE procedure; those not meeting normal distribution were transformed using natural logarithm, and those still not meeting normality by this method were evaluated by non-parametric statistics.

Variables with normal distribution were analyzed using the PROC GLM procedure, which evaluates non-balanced variables, testing for interactions between treatment (Cyclic or Fragment), and if these had a statistical effect on the variables. Means were evaluated using the Tukey’s test, using the LSMEANS procedure. Variables not meeting normal distribution were analyzed by the PROC NPAR1WAY procedure to evaluate the means by Wilcoxon and Kruskal-Wallis tests. Data are presented as means ± standard deviation. Differences P ≤ 0.05 were considered significant. Interactions were evaluated between horns, arteries, and days.

### Ethics committee approval

This study was carried out with a protocol approved by the Animal Ethical Use Committee from Universidade Federal do Rio Grande do Sul, Porto Alegre, Rio Grande do Sul, Brazil (protocol number 34572).

## Results

Biopsies from the ten mares were classified as Category I (70%) and Category IIA (30%).

The percentages of ciliated, secretory protruded and flattened cells, apical blebs, superficial secretion, intraglandular secretion, glandular diameter, glandular lumen, glandular density, and height of glandular epithelium did not differ between the contralateral horn to the corpus luteum and the ipsilateral horn (P > 0.05).

**Figure 2:**
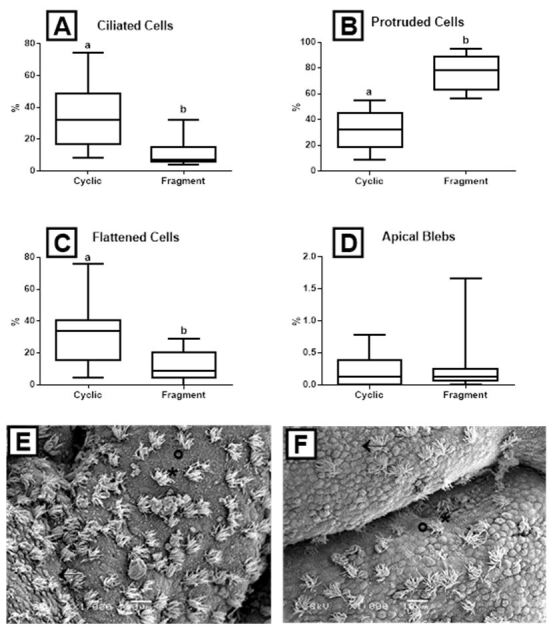
Box-plots (whiskers represent min to max) of (A) ciliated cells; (B) protruded cells; (C) flattened cells; and (D) apical blebs of the Cyclic and Fragment groups. Different letters (a, b) represent significant difference (*P* < 0.002); (E) Scanning electron micrograph (1,000x, bar = 10 µm) of the endometrium of the Cyclic group; (F) Scanning electron micrograph (1,000x, bar = 10 µm) of the endometrium of the Fragment group. Asterisks (*) indicate ciliated cells, black arrows indicate protruded cells, and circles show flattened cells.

The percentage of ciliated cells per field showed a significant decrease (P = 0.001) in the Fragment group (11.0 ± 8%) in comparison with the Cyclic group (33.7 ± 17%) (Figures2A, 2E, 2F). However, the percentage of protruded cells was higher (P = 0.002) in the Fragment group (76.9 ± 13%) than in the Cyclic group (32.9 ± 14%) (Figures 222F). In the infused mares, the percentage of flattened cells decreased (P = 0.001) (11.3 ± 9%) compared with cyclic mares (32.9 ± 17%) (Figures 222F). No difference was detected in apical blebs (P = 0.416) between the Cyclic and Fragment groups (Figure 2D).

In the cyclic mares, superficial secretion occupied a lower endometrial area per field (0.2 ± 0.1%), whereas in the infused mares (0.3 ± 0.1%), secretion was more abundant (P = 0.003) (Figures 333D). Likewise, in the Cyclic group, intraglandular secretion (1.3 ± 0.4 μm) was less abundant than in the Fragment group (3.2 ± 0.7 μm) (P < 0.001) (Figures333F).

**Figure 3:**
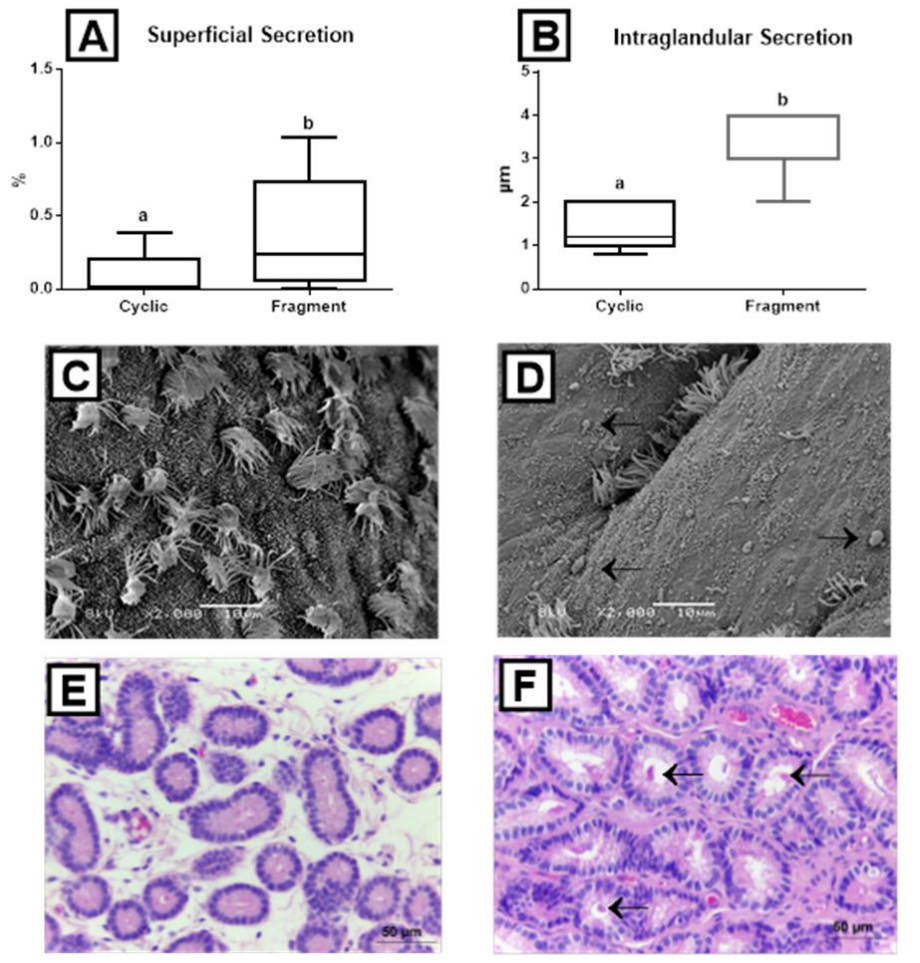
Box-plots (whiskers represent min to max) of (A) superficial secretion and (B) intraglandular secretion of the Cyclic and Fragment groups. Different letters (a, b) represent significant difference (*P*< 0.03); (C) (Cyclic group) Scanning electron micrograph (2,000x, bar = 10 µm) of the endometrium showing ciliated cells, protruded secretory cells, flattened secretory cells, and secretion; (D) (Fragment group) Scanning electron micrograph (2,000x, bar = 10 µm) of the endometrium showing ciliated cells, protruded secretory cells, flattened secretory cells, and secretion; (E) (Cyclic group) Histological section of endometrial glands (400x, bar = 20 µm); (F) (Fragment group) Histological section of endometrial glands (400x, bar = 20 µm). Black arrow shows superficial and intraglandular secretion.

A higher glandular diameter was observed in the Fragment group (50.4 ± 4 μm) than in the Cyclic group (41.9 ± 4 μm) (P < 0.001) (Figures 444D). Glandular lumen increased in the group infused with conceptus fragments (18.9 ± 3 μm), in comparison with the Cyclic group (9.4 ± 2 μm) (P < 0.001) (Figures 444D).

**Figure 4:**
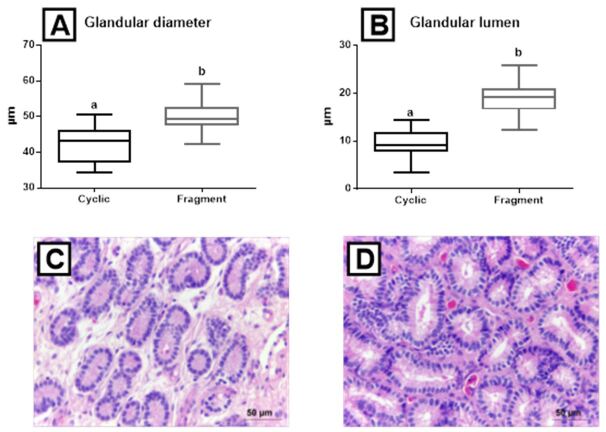
Box-plots (whiskers represent min to max) of (A) glandular diameter and (B) glandular lumen of the Cyclic and Fragment groups. Different letters (a, b) represent significant difference (*P* < 0.001); (C) (Cyclic group) histological section of endometrial glands (400x, bar = 20 µm); (D) (Fragment group) histological section of endometrial glands (400x, bar = 20 µm), equine endometrial tissues.

Glandular density did not differ between groups (P = 0.291), neither in the stratum compactum nor in the stratum spongiosum. The height of glandular epithelium and luminal epithelium were similar between Fragment and Cyclic groups (P = 0.501 and P = 0.099, respectively).

**Figure 5:**
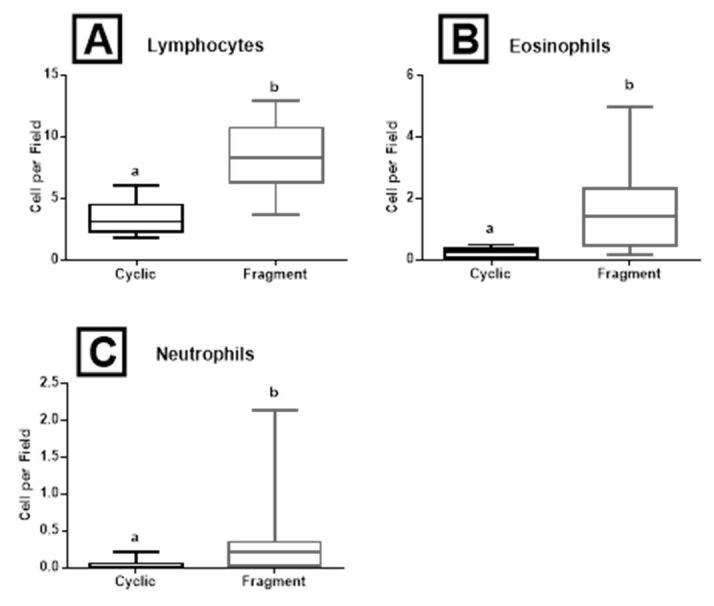
Box-plots (whiskers represent min to max) of (A) lymphocytes; (B) eosinophils; and (C) neutrophils of the Cyclic and Fragment groups in the equine endometrial tissues. Different letters (a, b) represent significant difference (*P* = 0.001).

The number of counted lymphocytes was higher (P < 0.001) in the Fragment group (8.3 ± 2 cells/field) than in the Cyclic group (3.4 ± 1 cells/field) (Figure 5A). Similarly, the number of eosinophils and neutrophils was higher (P < 0.001) in the Fragment group (1.5 ± 1.0 cells/field and 0.3 ± 0.3 cells/field respectively) in comparison with the Cyclic group (0.2 ± 0.1 cells/field and 0.04 ± 0.04 cells/field respectively) (Figures 55C). The presence of immune cells was similar in then stratum compactum and stratum spongiosum. There was neither influence nor interaction between horn and treatment.

**Figure 6:**
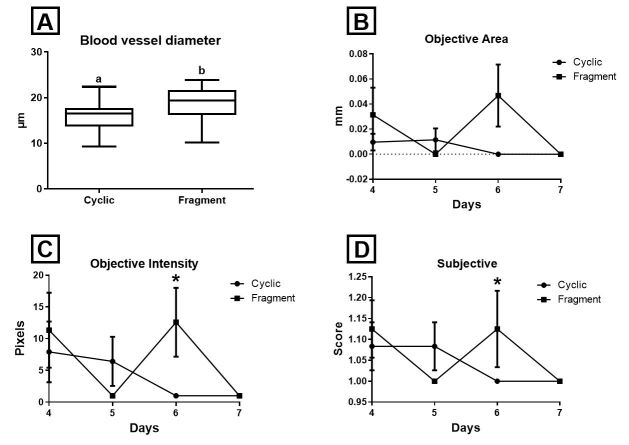
Box-plots (whiskers represent min to max) of (A) blood vessel diameter of the Cyclic and Fragment groups (7^th^ day). Different letters (a, b) represent significant difference (*P* = 0,022); (B) (4^th^ to 7^th^ day) Power Doppler objective endometrial vascularization area; (C) (4^th^ to 7^th^ day) Power Doppler objective intensity of endometrial vascularization; (D) (4^th^ to 7^th^ day) Subjective endometrial vascularization. Asterisks (*) represent significant difference (*P* < 0.05).

Histological analysis at the 7^th^ day after ovulation showed endometrial blood vessels with higher (P = 0.022) diameter in the Fragment group (18.5 ± 3 μm) than in the Cyclic group (15.9 ± 3 μm) (Figure 6A).

The blood-flow area did not differ (P = 0.06) between the Fragment and the Cyclic groups (Figure 6B). On day 6, the Fragment group presented higher blood flow intensity in the objective evaluation (P = 0.038) (Figure 6C) and a larger extent of colored areas in the subjective evaluation (P = 0.048) (Figure 6D) than the Cyclic group. At day 7, no differences (P > 0.05) were observed in the blood flow evaluated by objective and subjective methods between the Fragment and Cyclic groups. Objective and subjective measurements of uterine vascularization did not differ between the uterine body, the contralateral horn to the corpus luteum, and the ipsilateral horn (P > 0.05).

The RI, PI, and TAMV values measured by spectral Doppler for the two uterine arteries between days 0 and 7 after ovulation did not show differences between groups (P > 0.05) or interactions (P = 0.722) between arteries and group.

## Discussion

Proper utero-embryonic communication is essential for the development and maintenance of pregnancy. This crosstalk is orchestrated by a complex network of humoral interactions with local and systemic implications. In the present study, rapid endometrial changes were evident two days after the infusion of equine conceptus fragments in mares on the 5th-day post ovulation.

The 13-day-old concepti were selected due to higher content of proteins in the yolk sac (Smits et al., 2018), with one or more of these proteins probably being involved in the anti-luteolytic mechanism (McDowell et al.,1990) which prolongs the lifespan of the corpus luteum during the maternal recognition of pregnancy (Sharp et al., 1989). The proteins present in the conceptus tissues remain inactive with unfolded conformations during storage in low temperatures, resulting in active molecules after the thawing process (Garber Cohen et al., 2010). The days of fragments infusion (5^th^ day) and uterine biopsy (7^th^ day) were selected because it is the period where serum progesterone concentration reaches its peak, both in pregnant and cyclic mares (Camozzato et al., 2019).

During pregnancy, the presence of the conceptus in the uterus alters the secretion of histotroph and growth factors to create a more favorable uterine environment for early embryonic survival (Wilsher et al., 2011). The proteins and transcripts contained in the concepti fragments infused in the present study can induce these changes to produce a satisfactory milieu. These stimuli likely increase the endometrial vascular perfusion (Silva et al., 2011) and the production of histotroph from the endometrial glands (Bazer et al., 1979), altering gene expression (Merkl et al., 2010).

In the present study, the cellular profiles two days after the infusion of the conceptus fragments differ from those observed in the endometrium of cyclic mares on the 7^th^ day of diestrus. Cellular profiles of the infused mares show similarity with the findings observed in the pregnant mares at the 7^th^ day of pregnancy: loss of ciliated cells, presence of protruded cells, the existence of histotrophic material, and large glandular diameter (Camozzato et al., 2019). These modifications were probably induced by the protein content of the material placed into the uterus. Recently, proteins of the 13^th^ day equine conceptus with relevance in the embryo-maternal communication were identified (Smits et al., 2018).

Vessel diameter was higher in the infused mares in comparison to the cyclic mares. This increase in vessel diameter in the endometrial stroma is similar to that observed in pregnant mares in comparison with cyclic mares (Camozzato et al., 2019). The complex regulation of angiogenesis and the vascular remodeling process is fundamental for the success or failure of the maintenance of the pregnancy (Tayade et al., 2007). Conceptus secretory products stimulate vasodilatation and angiogenesis to increase uterine blood flow and substrate delivery to the pregnant uterus (Bazer et al., 1986). Several studies have identified genes and proteins involved in the regulatory systems of the angiogenesis process, such as the vascular endothelial growth factor receptor (VEGF) system, the angiopoietin family, different regulators of endothelial cells, and hypoxia-induced genes (Merkl et al., 2010; Klein and Troedsson, 2011b; Silva et al., 2011; Bastos et al., 2019). This remodeling of vascularization is likely to play a role in maternal support of conceptus’ growth, preparing the uterus for the prospective pregnancy and facilitating the exchange of gases and nutrients (Merkl et al., 2010; Klein, 2016).

An increased rise in local vascular perfusion was detected in infused mares in contrast with cyclic mares. These increments suggest that proteins of the conceptus fragments produced changes in the endometrial vascularization on day 6, although these alterations disappeared by the 7^th^ day, probably due to the limited amount of protein content, denaturation, and the lack of the live embryo or its motility, or be a result of readaptation of the tissue. Increment in endometrial vascular perfusion of pregnant mares has been reported since the 7^th^ day after ovulation in relation to non-pregnant mares (Nieto-Olmedo et al., 2018).

No differences were observed between cyclic mares and infused mares in the hemodynamics of the uterine arteries. This result may be due to the limited amount of proteins and therefore lack a more prolonged stimulus. However, in pregnant mares, conceptus promotes changes in the hemodynamics of the uterine arteries starting on the 11^th^ day after ovulation (Bollwein et al., 2003).

An increased number of immune cells was observed in the Fragment group. The increase of lymphocytes suggests an adaptive immunologic response from the uterus, caused by contact with proteins of the conceptus fragments, where lymphocytes may induce an immunological response, and eosinophils may be involved in moderating this response (Keenan et al., 1987; Martinez Pereira, 2016). Therefore, the conceptus produces inflammatory mediators that aid in the embryonic-maternal cross-talk, and avoid rejection (Pearson, 2002; Waclawik, 2011; Waclawik et al., 2017), and immune cells are mediators of this communication.

A neutrophil increase was observed in the endometrium of the Fragment group in contrast to the Cyclic group. These endometrial changes observed in the present study are probably not associated with inflammation. Mares with endometritis show luminal epithelial degeneration, damaged ciliated cells with holes in the surface, ulcers around the mouths of the glands, cellular debris, and neutrophilic migration (Ricketts et al., 1978). These cellular alterations were not observed in the present study. In a recent study in women, a role for maternal neutrophils in maintaining normal pregnancy through their interactions with T cells, exposed to progesterone and estriol, was detected resulting in a population of T cells that are regulatory and proangiogenic (Nadkarni et al., 2016)

The possibility of corpus luteum lysis due to cervical manipulation during the infusion procedure may be excluded. High pregnancy rates are obtained after cervical manipulation (> 70%) (Cuervo-Arango et al., 2018). Endometrium of mares after prostaglandin application or in estrus present large numbers of apical blebs and flattened superficial cells (Samuel et al., 1979) profiles that were not observed in the infused mares. Moreover, the presence of protruded cells, glandular secretion, and large glandular diameter observed in the infused mares has not been detected in mares in estrus.

## Conclusion

The observed results confirm the hypothesis that proteins present in a 13^th^ day equine conceptus fragments caused histological and vascular changes in the endometrium at the 7^th^ day after ovulation, two days after their infusion in comparison with the same cyclic mares on the 7^th^ day without manipulation. The proteins and transcripts contained in the conceptus fragments probably induced secretion of histotroph, a local vascular increase, and an immunologic response from the uterus, necessary for pregnancy establishment, however, it could be also caused by an inflammatory response. These alterations are similar to those observed in pregnant mares endometrium with a live conceptus on the 7^th^ day. The proteins of 13^th^ day conceptus fragments may be involved in the embryo-maternal communication and perhaps in the maternal recognition of pregnancy. Changes in glandular secretion likely modify endometrial fluid; therefore, the uterine fluids should be analyzed in further studies.
